# High-volume bilateral chylothorax presenting with hypoxemia and shock in a pediatric patient following tracheostomy revision: a case report

**DOI:** 10.1186/s13256-015-0721-6

**Published:** 2015-10-22

**Authors:** Aaron L Thatcher, Jane Yu, Kevin W Kuo

**Affiliations:** 1 Department of Otolaryngology, University of Michigan, 1500 E Medical Center Drive, Ann Arbor, MI 48109 USA; 2 Department of Pediatrics and Communicable Diseases, C.S. Mott Children’s Hospital, University of Michigan, 1500 E Medical Center Drive, Ann Arbor, MI 48109 USA

**Keywords:** Bilateral chylothorax, Complication, Neck dissection, Pediatric, Tracheoplasty

## Abstract

**Introduction:**

Chylothorax is a rare complication of surgical neck dissection. This is the first reported pediatric case of bilateral chylothorax following cervical surgery and the first to occur after tracheoplasty. Chylothorax can lead to significant complications, including hypoxemia and shock, and requires timely treatment. This case report discusses the clinical presentation, diagnosis, and treatment of our patient and reviews possible pathophysiologic mechanisms to explain the development of postoperative bilateral chylous effusions.

**Case presentation:**

An 18-month-old white baby girl with a complex past medical history including choanal atresia, atrioventricular septal defect, failure to thrive, developmental delay, and tracheostomy dependence developed significant hypoxemia and shock following a routine tracehostomy revision. She was subsequently found to have developed massive bilateral chylothorax, requiring escalation of mechanical ventilation, thoracostomy tube drainage, vasoactive support, and eventual surgical ligation of her thoracic duct.

**Conclusions:**

Massive bilateral chylothorax is a rare but potentially life-threatening complication following tracheoplasty. Clinicians caring for this patient population postoperatively should be aware of this potential complication and its management.

## Introduction

Chylothorax is the accumulation of chyle in the pleural space due to disruption or obstruction of the thoracic duct. Chyle is lymphatic fluid absorbed from the intestinal tract; it is rich in triglycerides, lymphocytes, immunoglobulins, and other small-molecular-weight proteins. Chyle is transported from the intestinal tract to the upper central venous system by the thoracic duct. The thoracic duct begins at the cisterna chyli located at the level of the first or second lumbar vertebra. It enters into the thoracic cavity via the aortic hiatus and continues to ascend in the posterior mediastinum between the aorta and the azygous vein. At the fifth thoracic vertebral level, the duct crosses over from the right side to the left and continues its course until it reaches and empties into venous circulation. The termination point is highly variable but most commonly occurs at the junction of the left internal jugular and subclavian veins.

The etiology of chylothorax can be divided into four categories: malignancy, trauma, idiopathic, and miscellaneous. Traumatic chylothorax most commonly occurs as a complication of thoracic surgery [1]. In the pediatric population, traumatic chylothorax is associated most frequently with cardiothoracic surgery for congenital heart disease with a reported incidence of 8.9% at this institution [2]. Chylothorax following neck surgery, on the other hand, is rare. To date, only 22 cases of bilateral chylothorax as a complication of neck dissection have been reported in the English literature, all described in adult patients. These have occurred almost exclusively in the setting of radical neck dissection for various malignancies. Typical management has included thoracentesis, thoracostomy tube drainage, low fat diet or nil by mouth (NPO) and, in rare instances, thoracic duct ligation.

Diagnosis of chylothorax usually begins with radiographic imaging revealing a pleural effusion on chest X-ray after development of respiratory distress. The diagnosis of chylothorax requires laboratory evaluation of pleural fluid in order to differentiate it from other types of pleural effusion. The presence of chylomicrons is diagnostic. Table 1 lists pleural fluid laboratory characteristics of chylothorax [3].

**Table 1:** Pleural fluid laboratory characteristics of chylous effusion

Appearance	Variable (milky, sanguineous, serous)
Lipoprotein analysis	Presence of chylomicrons
Cell count	Lymphocyte predominance
pH	Alkaline
Triglycerides	Typically >110mg/dL
Lactate dehydrogenase	Exudative range
Protein	2–3mg/dL

The management approach for chylothorax is variable. Management initially involves dietary modification to reduce chyle flow as well as somatostatin analogs to reduce lymphatic flow. Drainage of pleural fluid may be necessary if it causes respiratory distress. The patient must be monitored for sequelae from chyle loss and abnormalities corrected. Conservative management is often successful but surgical management is occasionally necessary. A systematic trial of conservative treatment for 7 to 10 days followed by tiered surgical intervention can minimize the length of Intensive Care Unit (ICU) stay, hospital stay, and length of medical device use [2].

Here we present the first pediatric case of bilateral chylothorax after cervical surgery and the first case of chylothorax following tracheostoma revision.

## Case presentation

An 18-month-old white baby girl who is tracheostomy and ventilator dependent underwent cartilage graft tracheoplasty and developed the postoperative complication of bilateral chylothorax. Her past medical history is complex and includes choanal atresia, atrioventricular septal defect, complete heart block, congestive heart failure, failure to thrive, tracheobronchomalacia, developmental delay, and hypothyroidism. Her past surgical history is significant for bilateral choanal atresia repair, patent ductus arteriosus (PDA) ligation at age 1 month, partial atrial septal defect/ventricular septal defect (ASD/VSD) closure at age 2 months, subsequent cardiac pacemaker implantation at age 2 months complicated by implant infection requiring explantation at age 3 months. Due to heart failure and tracheobronchomalacia, she underwent tracheostomy at age 3 months with an intraoperative note of continuity of the cervical field with the mediastinal surgical fields. Her pacemaker was replaced at age 4 months and was complicated by left chylothorax, which resolved with chest tube drainage and NPO diet with parenteral nutrition. Other significant procedures include gastrojejunostomy (GJ) tube placement at age 5 months complicated by small bowel perforation requiring open repair.

She was first discharged home after birth at age 9 months with a cuffed tracheostomy tube, ventilator dependent, and gastrostomy tube dependent. During out-patient follow-up, evaluation of her tracheostomy site revealed significant inferior tracheostoma breakdown with difficulty maintaining a seal for adequate airway pressures as well as imminent innominate artery exposure. She was also noted to have significant hearing loss due to chronic otitis media with effusion. Pediatric surgery separately evaluated her gastrostomy tube with leakage noted around the site. Therefore tracheoplasty, bilateral myringotomy and tympanostomy tube insertion, and gastrostomy tube revision were scheduled.

She underwent combined procedures with otolaryngology and pediatric surgery under a single anesthetic. Bilateral myringotomy and tympanostomy, direct laryngoscopy, bronchoscopy, and anterior cartilage graft tracheoplasty were performed by otolaryngology. Pediatric surgery performed gastrocutaneous fistula closure and Broviac catheter placement. During the skin incision for tracheoplasty, an obvious chyle leak was encountered just deep to the skin and 2mm left of the tracheostoma. This was controlled using a figure-of-eight stitch with a 2–0 silk suture. No further chyle leak was detected during the remainder of the case.

**Fig. 1:**
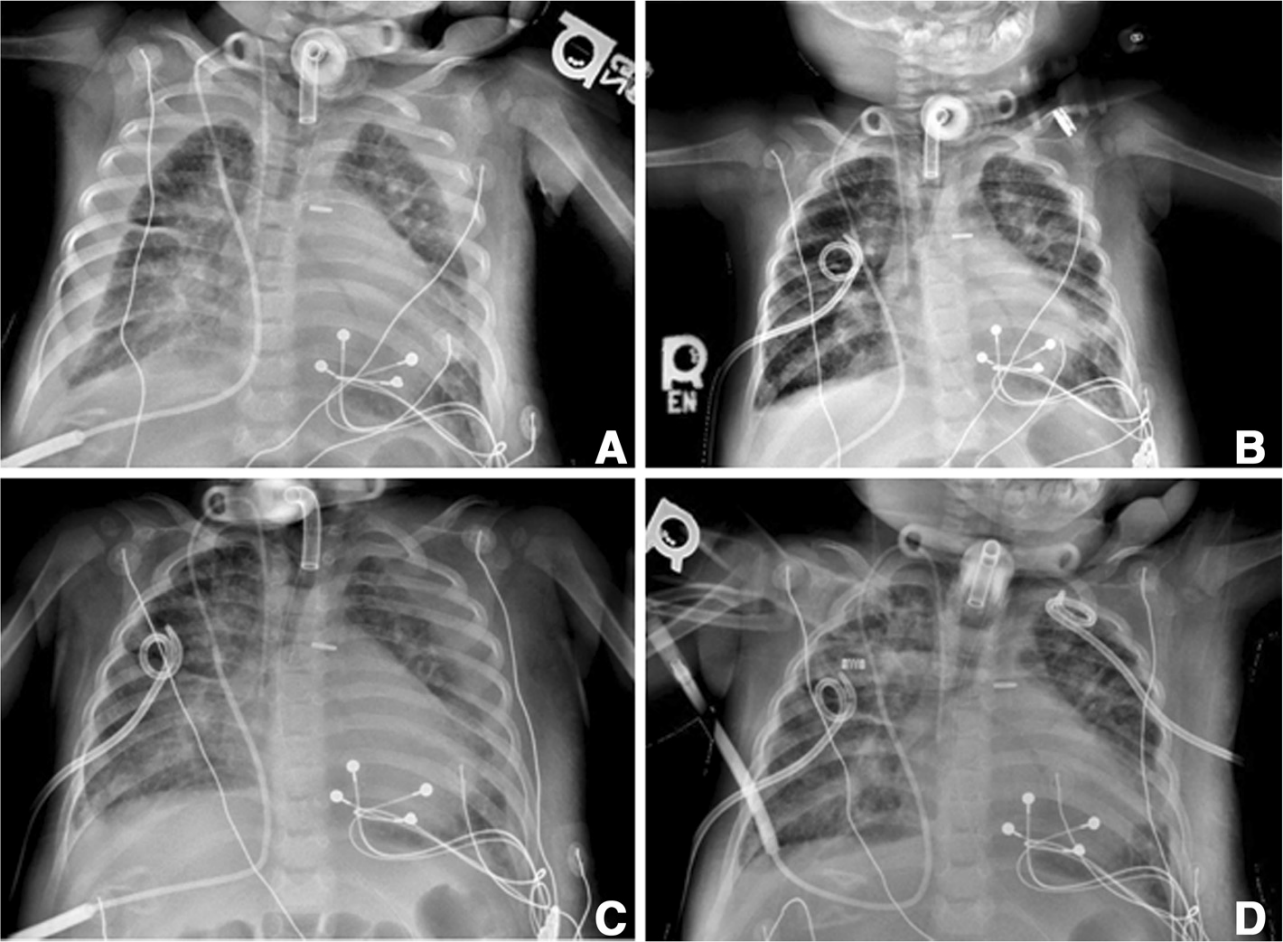
Chest X-ray images of pleural effusions. **a** Bilateral pleural effusions noted on postoperative day 3. **b** Following right chest tube placement and left thoracentesis on postoperative day 3. **c** Reaccumulation of left pleural effusion on postoperative day 4. **d** Following left chest tube placement on postoperative day 4

Postoperatively she was admitted to the pediatric ICU. On the third postoperative day, she developed worsening hypoxemia and respiratory distress. The ventilator was escalated from home settings of peak inspiratory pressure (PIP) 24cmH_2_O, positive end-expiratory pressure (PEEP) 8cmH_2_O, and fraction of inspired oxygen (FiO_2_) 21% to PIP 30cmH_2_O, PEEP 12cmH_2_O, and FiO_2_ 45%. A chest X-ray revealed a large right pleural effusion and a smaller left pleural effusion (Fig. 1). Given clinical decompensation, the decision was made to drain the effusions. An 8.5 French pigtail drain was inserted in the right pleural space and thoracentesis was performed on the left pleural effusion. A total of 130mL of milky fluid was drained from the right pleural space prior to placement of a chest tube drainage system. A total of 30mL of milky fluid was drained from the left. The left effusion accumulated over the next 24 hours prompting placement of a chest tube on postoperative day 4. Pleural fluid laboratory studies were sent and were diagnostic for a chylous effusion (Table 2). Other diagnostic considerations included hemothorax, simple effusion secondary to heart failure, or empyema secondary to pneumonia.

**Table 2:** Pleural fluid laboratory results

Appearance	Opaque
Color	Yellow
Red blood cell count	<3000 cells/mm^3^
Leukocyte count	2321 cells/mm^3^
Neutrophils	14%
Lymphocytes	70%
Histiocytes	7%
Plasma cells	9%
pH	8.49
Triglycerides	261mg/dL
Lactate dehydrogenase	124 IU/L
Protein	3.1gm/dL

The patient proceeded to have significant chest tube output particularly from the right side (>130mL/kg/day). Output was significant enough to cause hemodynamic instability. Four 10ml/kg fluid boluses with either 0.9% normal saline or 5% albumin were administered for hypotension during the first 24 hours post-chest tube placement. Due to continued significant chest tube drainage and subsequent fluid refractory hypotension, vasoactive support was provided with an epinephrine infusion for 3 days. In order to prevent further hemodynamic instability from chest tube losses, ml per ml volume replacement of chest tube output with 5% albumin was started.

She met our institution’s criteria for high-output chylothorax (>20mL/kg/day), and treatment was initiated according to protocol [2]. An NPO diet with parenteral nutrition and octreotide infusion (3mcg/kg/hour) were ordered for 8 days. Following this, the left chylothorax resolved but right chylous output remained high. Therefore, we elected to proceed with thoracic duct ligation. Ligation was performed via right thoracotomy on postoperative day 12. Octreotide was discontinued postoperatively while NPO status with parenteral nutrition was continued for 3 more days. An enteral diet was introduced 5 days post-procedure with no further chyle leak. All chest tubes were subsequently removed and she was discharged home 10 days after thoracic duct ligation. She continued on a medium-chain fatty acid diet for 6 weeks following thoracic duct ligation. She has had no further complications from her chylothorax.

## Discussion

This is the first known report of bilateral chylothorax after neck surgery in a pediatric patient as well as the first known report of chylothorax following tracheostoma revision.

Given the anatomical structure of the thoracic duct, postsurgical chylothorax tends to occur unilaterally. Thoracic duct injury below the fifth thoracic vertebral body usually results in a right-sided pleural effusion while injury above usually results in a left-sided effusion [4]. Although not uncommon following thoracic surgery, chylothorax following neck dissection is rare with a reported incidence of 1 to 2% [5]. When it occurs, it usually results in a left-sided effusion.

Treatment recommendations for chylothorax are variable [5–7] but prudent management should include low-risk nonoperative treatments initially. Aside from identification and treatment of the underlying cause, the initial therapy should involve dietary modification to reduce chyle flow using a low fat or NPO diet. If dietary modification is not adequate, then administration of somatostatin or an analog such as octreotide can be considered. Somatostatin works by directly acting on somatostatin receptors reducing lymphatic fluid production, thereby reducing chyle flow. In cases where cardiorespiratory status is compromised, thoracentesis with thoracostomy tube placement should be performed and appropriate ventilatory support provided. Common clinical practice is a trial of conservative management with dietary modification and/or octreotide for approximately 1 to 2 weeks. If output remains high after this time or spontaneous healing is unlikely, consultation with a cardiothoracic surgeon should occur soon after diagnosis to determine indications for thoracic duct ligation. Early surgical intervention in cases of high-flow chyle leak failing medical therapy can reduce hospital stay, ICU stay, and the use of intensive care medical devices [2].

Aside from direct management of chyle leak, patients require close monitoring for complications of chyle loss. Chyle contains triglycerides, small-molecular weight proteins (albumin, immunoglobulin), and lymphocytes. Therefore, patients are at risk for malnutrition, hypovolemia, metabolic and electrolyte abnormalities, and immunodeficiency. If enteral nutrition is possible, a fat-free diet can be instituted using a medium-chain triglyceride formula. Medium-chain triglycerides are saturated fatty acids that are of 8 to 12 carbon lengths and their use is indicated in chylothorax because they are directly taken up by portal circulation, bypassing lymphatic circulation. Response to a medium-chain triglyceride diet should be seen within the first week with full resolution of the chylothorax by the end of the second week (goal <10mL/kg/day) [8]. If enteral dietary modification fails or is not an option due to severity of chylothorax, then full NPO status is required with total parenteral nutrition and lipids to maintain nutritional status.

Hypovolemia can result if a chyle leak is significant enough from both general volume loss and albumin loss [9]. Hypoalbuminemia can exacerbate hypotension due to third spacing that occurs secondary to reduced oncotic pressure. Intravascular resuscitation with fluid boluses may be necessary and repletion of ongoing chyle loss with ml per ml replacement fluid should be considered. Albumin levels should be followed closely and replacement with concentrated albumin considered for level <3g/dL, specifically in critically ill patients [10, 11]. Electrolyte and acid–base derangements can develop with hyponatremia, hypocalcemia, and metabolic acidosis of particular concern [12]. Lastly, secondary immunodeficiency can occur due to loss of both immunoglobulins and lymphocytes [13]. Routine immunoglobulin replacement with immunoglobulin administered intravenously (IVIG) for hypogammaglobulinemia has not demonstrated a protective effect for pediatric patients with chylothorax [14] but should be considered for patients who have additional risk factors for serious infection.

While our patient did have a cervical chyle leak identified and repaired intraoperatively, the development of massive bilateral chylothorax following cervical surgery remains puzzling. There was no intrathoracic injury to the thoracic duct nor was there injury to the pleura evidenced by the absence of pneumothorax. Two pathophysiologic mechanisms have been suggested as the etiology of chylothorax after cervical surgery. The first is direct leakage of chyle into the mediastinum from the thoracic duct. Leakage of chyle results in an inflammatory reaction that allows penetration of chylous fluid from the mediastinum into the pleural space through the inflamed pleura. The second is ligation of the distal thoracic duct, which leads to increased hydrostatic pressure within the fragile lymphatic vessels. The build-up of pressure within the thoracic duct, combined with negative intrathoracic pressure, leads to either rupture or non-traumatic leakage of chyle into the mediastinum and pleural spaces [3, 10]. Either of these mechanisms could have contributed to the development of bilateral chylothorax in our patient.

## Conclusions

In summary, chylothorax is a rare complication of neck surgery. This is the first reported pediatric case of bilateral chylothorax following cervical surgery and the first to occur after tracheoplasty. Clinicians caring for patients after neck dissection should be aware of this rare but significant complication and the various strategies for effective treatment.

## Consent

Written informed consent was obtained from the patient’s mother for publication of this case report and accompanying images. A copy of the written consent is available for review by the Editor-in-Chief of this journal.

## Abbreviations

FiO_2_: Fraction of inspired oxygen
ICU: Intensive Care Unit
NPO: Nil by mouth
PEEP: Positive end-expiratory pressure
PIP: Peak inspiratory pressure
